# Higher Platelet-to-Lymphocyte Ratio Increased the Risk of Sarcopenia in the Community-Dwelling Older Adults

**DOI:** 10.1038/s41598-017-16924-y

**Published:** 2017-11-30

**Authors:** Fang-Yih Liaw, Ching-Fu Huang, Wei-Liang Chen, Li-Wei Wu, Tao-Chun Peng, Yaw-Wen Chang, Tung-Wei Kao

**Affiliations:** 1 Division of Geriatric Medicine, Department of Family and Community Medicine, Tri-Service General Hospital, Taipei, Taiwan; 2 Department of Dermatology, Taoyuan Armed Forces General Hospital, Taoyuan, Taiwan; 3 National Defense Medical Center, Taipei, Taiwan

## Abstract

The platelet-to-lymphocyte ratio (PLR) has been extensively studied in oncologic diseases. However, the correlation between PLR and sarcopenia remains unknown. In this cross-sectional analysis, we enrolled 3,671 non-institutionalized individuals from the National Health and Nutrition Examination Survey (NHANES) III (1988–1994) aged ≥60 years and whose complete blood counts (CBCs), body composition measurements, and related demographic information was available. Skeletal muscle mass was assessed using a previously published equation (including age, sex, height, and bioelectrical impedance analysis). PLR values were estimated based on laboratory data. Multiple linear and logistic regression analyses, quartile-based stratified odds ratio comparisons, and trend tests were performed. Elevations in serum PLR values were significantly associated with sarcopenia status and negatively associated with skeletal muscle index. After additionally adjusting for other covariates, the significant negative correlation remained; moreover, participants with highest serum PLR values (≥155) had 2.36 times greater risk of sarcopenia than those with lowest PLR values (<90; odds ratio (OR) = 2.36; 95% confidence interval (CI): 1.21–3.31; p < 0.01). Higher PLR levels are associated with a greater risk of sarcopenia in geriatric populations. Thus, PLR as an inexpensive and easily measurable parameter can be considered as an inflammatory biomarker for sarcopenia.

## Introduction

The platelet-to-lymphocyte ratio (PLR) is a simple blood test that reflects variations in platelet and lymphocyte levels. Elevations in PLR are associated with poor prognosis in various oncologic diseases. As platelets and lymphocytes play a key role in the inflammatory process, PLR is regarded as an important indicator of systemic inflammation and also significant in multiple non-oncologic diseases, such as cardiovascular disease^1^, diabetes mellitus^2^, and autoimmune diseases^3^. Inflammatory cytokines reportedly induce muscle wasting and ultimately alter protein catabolism, as well as inhibit muscle synthesis. High levels of inflammatory cytokines are negatively related to muscle strength and mass^4^.

Sarcopenia, the age-dependent loss of muscle function and mass, is a common geriatric syndrome that is associated with multiple adverse health outcomes. High levels of serum inflammatory markers are associated with sarcopenia, with chronic inflammation playing a role in this disorder^5^. Given their respective relationships with inflammatory status, sarcopenia and PLR may be correlated; however, no study has examined the relationship between PLR and sarcopenia. In the present study, we examined the relationship between clinical PLR values in geriatric patients with sarcopenia. Moreover, we examined the utility of PLR as a novel biomarker of sarcopenia.

## Results

### Participants

Altogether, 39,695 participants were included in the National Health and Nutrition Examination Survey (NHANES) III dataset between 1988 and 1994, with 13,454 participants having complete anthropometric and bioelectrical impedance data for the estimation of the skeletal muscle index (SMI). We included participants aged ≥60 years (n = 4,087) and excluded participants with non-skin cancer (n = 319), as well as those taking steroids and immunomodulators (n = 16). Participants lacking data from the laboratory measurements, clinical examinations, and household interviews were also excluded (n = 81). Finally, 3,671 participants were examined.

### Characteristics of the study population

The characteristics of all the participants stratified by the sarcopenia status are summarized in Table 1. Of the 3,671 participants recruited in the study, 1,813 (49.3%) were male. The prevalence of class I sarcopenia and class II sarcopenia were 61.8% and 19.7% in male patients, and 28.8% and 14.8% in female patients, respectively. Participants with sarcopenia were older, had higher PLR levels, were more likely to be current smokers, and had a lower prevalence of metabolic syndrome (MetS) compared to the control group.

**Table 1:** Characteristics of the study participants.

Variables	Male (n = 1813)				Female (n = 1858)			
	No sarcopenia (n = 335)	Class I sarcopenia (n = 1121)	Class II sarcopenia (n = 357)	*P* value	No sarcopenia (n = 1046)	Class I sarcopenia (n = 536)	Class II sarcopenia (n = 276)	*P* value
**Continuous variables**								
Age (years), mean (SD)	68.02 (6.69)	70.77 (7.72)	73.35 (8.15)	<0.001	70 (7.17)	73.35 (8.43)	74.04 (8.53)	<0.001
Platelet-lymphocyte ratio, mean (SD)	116.39 (40.75)	127.24 (54.64)	136.08 (58.46)	<0.001	125.14 (44.43)	140.12 (54.83)	147.16 (61.42)	<0.001
Neutrophil-lymphocyte ratio, mean (SD)	2.18 (1.22)	2.29 (1.59)	2.52 (1.41)	0.196	2.09 (1.27)	2.24 (1.24)	2.33 (1.58)	0.277
C-reactive protein (mg/dL), mean (SD)	0.53 (0.86)	0.5 (0.92)	0.61 (0.96)	0.165	0.63 (1.06)	0.56 (1.13)	0.55 (0.917)	0.361
Plasma fibrinogen (mg/dL), mean (IQR)	576.1 (87)	569.1 (94)	670.6 (118)	0.521	519.6 (100)	666.47 (101)	643.7 (129)	0.115
Serum uric acid (mg/dL), mean (SD)	6.18 (1.38)	6.09 (1.41)	6.02 (1.43)	0.319	5.43 (1.49)	5.02 (1.34)	5.21 (1.58)	<0.001
Serum bilirubin (mg/dL), mean (SD)	0.64 (0.25)	0.68 (0.33)	0.65 (0.29)	0.057	0.52 (0.26)	0.52 (0.26)	0.49 (0.17)	0.124
**Categorical variables**								
Non-Hispanic white, *n* (%)	176 (52.5)	654 (58.3)	198 (55.5)	0.623	597 (57.1)	350 (65.3)	162 (58.7)	0.058
Coronary heart disease, *n* (%)	40 (11.9)	143 (12.8)	46 (12.9)	0.459	76 (7.3)	36 (6.7)	20 (7.2)	0.099
Stroke, *n* (%)	20 (6)	58 (5.2)	28 (7.8)	0.054	55 (5.4)	27 (5.1)	29 (10.6)	0.006
Metabolic syndrome, *n* (%)	217 (64.8)	546 (48.7)	145 (40.6)	<0.001	746 (71.3)	284 (53)	139 (50.4)	<0.001
Alcohol drinking, *n* (%)	96 (59.3)	268 (50.9)	83 (52.5)	0.062	266 (57.3)	140 (57.6)	67 (54.5)	0.954
Smoking, *n* (%)	41 (17.5)	211 (26.4)	91 (35.3)	<0.001	110 (30.2)	76 (39.2)	45 (43.3)	0.016

Table 2 illustrates the data divided into 4 groups based on PLR level quartiles. The highest PLR level group had a higher prevalence of sarcopenia, a lower SMI and a slower gait speed; moreover, this group was older, had a higher C-reactive protein (CRP) and lower albumin levels. The highest PLR quartile group had a greater proportion of non-Hispanic Caucasians and women (p < 0.05) compared to the other quartile groups.

**Table 2:** Characteristics of study participants classified by quartiles of PLR.

	Quartiles of PLR				
	Q1 (<95) n = 926	Q2 (95 to <120) n = 834	Q3 (120 to <155) n = 941	Q4 (≥155) n = 925	*P* value
**Continuous variables**					
Age, years	70.77 (7.81)	70.72 (7.61)	71.23 (8.14)	72.12 (7.94)	<0.001
C-reactive protein (mg/dL), mean (SD)	0.49 (0.6)	0.47 (0.78)	0.53 (0.77)	0.76 (1.55)	<0.001
Plasma fibrinogen (mg/dL), mean (IQR)	570.21 (96)	548.01 (92)	612.88 (102)	572.75 (123)	0.839
Serum uric acid(mg/dL), mean (SD)	5.88 (1.51)	5.74 (1.47)	5.62 (1.44)	5.52 (1.61)	0.027
Serum bilirubin (mg/dL), mean (SD)	0.61 (0.32)	0.6 (0.29)	0.58 (0.27)	0.56 (0.26)	0.098
Serum creatinine (mg/dL), mean (SD)	1.2 (0.36)	1.19 (0.48)	1.2 (0.51)	1.19 (0.56)	0.983
Serum albumin (mg/dL), mean (SD)	4.05 (0.33)	4.08 (0.32)	4.06 (0.35)	4.01 (0.36)	<0.001
serum vitamin D (nmol/L), mean (SD)	56.8 (19.35)	56.45 (19.26)	56.28 (19.89)	56.4 (20.92)	0.946
**Skeletal muscle index (kg/m** ^**2**^ **)**					
Male	9.6 (2.21)	9.06 (2.62)	9.22 (2.24)	8.98 (2.33)	<0.001
Female	6.95 (1.96)	6.97 (1.77)	6.5 (2)	6.41 (1.94)	<0.001
Gait speed (m/s), mean (SD)	0.72 (0.2)	0.73 (0.2)	0.72 (0.21)	0.7 (0.2)	0.03
**Categorical variables** ^b^					
Male, *n* (%)	506 (54.6)	416 (49.9)	471 (50.1)	396 (42.8)	<0.001
Non-Hispanic white, *n* (%)	799 (76.7)	692 (77.9)	829 (79.4)	844 (82.4)	0.003
Coronary heart disease, *n* (%)	98 (10.6)	93 (11.2)	86 (9.1)	87 (9.4)	0.075
Stroke, *n* (%)	56 (6)	55 (6.6)	55 (5.8)	55 (5.9)	0.807
Metabolic syndrome, *n* (%)	540 (58.3)	477 (57.2)	525 (55.8)	505 (54.6)	0.398
Alcohol drinking, *n* (%)	240 (57.4)	215 (56.6)	223 (51.1)	227 (55.1)	0.538
Smoking, *n* (%)	190 (35.8)	130 (29.8)	140 (28.3)	105 (22.2)	<0.001
Sarcopenia class I, *n* (%)	411 (44.7)	359 (43.4)	435 (46.9)	418 (45.5)	0.586
Sarcopenia class II, *n* (%)	124 (13.5)	131 (15.8)	155 (16.7)	209 (22.8)	<0.001

### PLR levels and sarcopenia

In the linear model, PLR negatively associated with the SMI (Table 3). The β coefficient, representing the change in the SMI for each 1- standard deviation (SD) increase in PLR value, was −0.003 (p = 0.003) in men and −0.005 (p < 0.001) in women. After adjusting for age and race-ethnicity (Model 1), the β coefficient was −0.002 (p = 0.012) in men and −0.004 in women (p < 0.001). For both sexes, after additionally adjusting for other covariates in Model 2, 3 and 4, the β coefficients were similar and the negative correlations remained.

**Table 3:** Multivariate linear regression model using skeletal muscle index as the dependent variable, and PLR levels and other factors indicated as the independent variables.

Models	Male		Female	
	β (S.E.)	*P* value	β (S.E.)	*P* value
Unadjusted	−0.003 (0.001)	0.003	−0.005 (0.001)	<0.001
Model 1	−0.002 (0.001)	0.012	−0.004 (0.001)	<0.001
Model 2	−0.003 (0.001)	0.019	−0.004 (0.001)	<0.001
Model 3	−0.002 (0.001)	0.014	−0.004 (0.001)	<0.001
Model 4	−0.002 (0.001)	0.014	−0.004 (0.001)	<0.001

Adjusted covariates: Model 1 = age, race-ethnicity. Model 2 = Model 1 + (stroke, heart disease, metabolic syndrome + current smoker). Model 3 = Model 2 + (white blood cell count, haemoglobin, C-reactive protein, fibrinogen, uric acid, bilirubin, creatinine, albumin, vitamin D). Model 4 = Model 3 + gait speed. S.E. = standard error.

Table 4 shows that participants with a higher PLR were at higher risk of sarcopenia (all, p < 0.05). In the unadjusted analysis, the odds ratio (OR) of predicted sarcopenia, for each increase in PLR value, was 1.004 (p < 0.001). After additional adjustment, the OR of predicted sarcopenia with each PLR value increase was 1.007 (p < 0.001). Collectively, these results suggest that increases in PLR are associated with a higher risk of sarcopenia.

**Table 4:** Association between PLR level and sarcopenia.

Models	OR (95% CI)	*P* value
Unadjusted	1.004 (1.003–1.006)	<0.001
Model 1	1.006 (1.005–1.009)	<0.001
Model 2	1.006 (1.005–1.008)	<0.001
Model 3	1.007 (1.005–1.009)	<0.001
Model 4	1.007 (1.005–1.009)	<0.001

Adjusted covariates: Model 1 = age, gender, race. Model 2 = Model 1 + (stroke, heart disease, metabolic syndrome + current smoker). Model 3 = Model 2 + (white blood cell count, haemoglobin, C-reactive protein, fibrinogen, uric acid, bilirubin, creatinine, albumin, vitamin D). Model 4 = Model 3 + gait speed. OR, odds ratio; CI, confidence interval. OR = odds ratio; CI = confidence interval.

The association between PLR level quartiles and elderly patients with sarcopenia is presented in Table 5. On multiple logistic regression analysis, participants with higher PLR levels exhibited a greater risk of sarcopenia (p < 0.05). In the unadjusted analysis, the OR of sarcopenia in cases in the Q3 and Q4 PLR groups were 1.25 and 1.72, respectively (p for trend, <0.001). After additional adjustment, the OR of sarcopenia in the cases in the Q4 PLR group was 2.36 (p for trend, <0.001).

**Table 5:** Association between serum PLR level quartiles and sarcopenia.

Models	Quartile of serum PLR levels								*P* for trend
	Q1		Q2		Q3		Q4		
	OR	*P* value	OR (95% CI)	*P* value	OR (95% CI)	*P* value	OR (95% CI)	*P* value	
Unadjusted	1.00 (reference)	—	1.08 (0.89–1.30)	0.42	1.25 (1.04–1.50)	0.015	1.72 (1.42–2.07)	<0.001	<0.001
Model 1	1.00 (reference)	—	1.20 (0.97–1.47)	0.09	1.40 (1.14–1.71)	0.001	2.24 (1.82–2.77)	<0.001	<0.001
Model 2	1.00 (reference)	—	1.19 (0.96–1.47)	0.12	1.36 (1.11–1.67)	0.004	2.21 (1.78–2.74)	<0.001	<0.001
Model 3	1.00 (reference)	—	1.19 (0.96–1.48)	0.11	1.39 (1.13–1.72)	0.002	2.36 (1.87–2.96)	<0.001	<0.001
Model 4	1.00 (reference)	—	1.18 (0.95–1.48)	0.12	1.39 (1.12–1.48)	0.002	2.36 (1.88–2.96)	<0.001	<0.001

Adjusted covariates: Model 1 = age, gender, race. Model 2 = Model 1 + (stroke, heart disease, metabolic syndrome, current smoker). Model 3 = Model 2 + (white blood cell count, haemoglobin, C-reactive protein, fibrinogen, uric acid, bilirubin, creatinine, albumin, vitamin D). Model 4 = Model 3 + (gait speed) ‡OR indicates odds ratio of sarcopenia comparing each subject in the upper three quartiles of serum PLR to those in the lowest quartile. OR = odds ratio; CI = confidence interval.

## Discussion

Complete blood counts (CBCs) are widely used to evaluate the general condition of patients. High platelet counts are signs of inflammation, whereas low lymphocyte counts indicate physiological stress and poor health^6^. PLR, an immune response-related indicator, is an objective and useful biomarker for evaluating subclinical inflammation in the peripheral blood^7^. A previous study showed that the effect of PLR on mortality was independent of the platelet or lymphocyte count alone^8^. There are 2 possible explanations for the superiority of PLR to either individual platelet or lymphocyte counts. First, PLR is more stable than the absolute lymphocyte or platelet counts, as several physiological and pathological conditions could alter these cell counts, including over-hydration, dehydration, and blood specimen handling. Second, PLR is a marker of 2 predictors that are inversely-related^8^. PLR is also less affected by subjective factors. Compared to erythrocyte sedimentation rate, CRP, other inflammation cytokines, and imaging, the measurement of PLR does not require additional cost. In this cross-sectional study, we found that PLR levels were greater in patients with sarcopenia than in those without sarcopenia. Thus, we found that PLR, an easily measurable and available laboratory parameter, was correlated with sarcopenia severity.

In the present study, the prevalence of class I and class II sarcopenia was among male and female patients was consistent with a previous study^9^. Sarcopenia is caused by age-related hormonal changes as well as changes in the inflammatory pathways, including elevation in the inflammatory cytokine levels^10^. Schrager *et al*. suggested that proinflammatory cytokines, such as interleukin (IL)-6, IL-18, CRP, and tumor necrosis factor (TNF)-α, play a critical role in the progression of sarcopenic obesity^11^. In the Health, Aging, and Body Composition Study, Schaap and colleagues assessed 2,177 geriatric subjects over 5 years to evaluate the association between the serum levels of inflammatory markers and loss of muscle mass and strength; they observed that higher levels of inflammatory markers were markedly associated with a greater 5-year decline in the thigh muscle area^12^. Sarcopenia is known as a key physiologic component of frailty, whereas frailty itself is related to changes in inflammation and coagulation^13^. Moreover, increasing age is associated with elevated platelet aggregability^14^. Therefore, both inflammation and platelet elevation are considered to play roles in the development of frailty. The Cardiovascular Health Study^15^ in 4735 community-dwelling geriatric adults evaluated the relationship between frailty status and physiological measures, including CRP, D-dimer, and factor VIII. Thus, it can be postulated that frailty is partly characterized by increased age-related inflammation and in the elevation in the levels of blood clotting markers.

An elevated PLR level is found to be a novel inflammation marker not only in various oncologic disorders, but also in non-oncologic disorders. Accumulating evidence suggests that chronic or systemic inflammation is associated with increased PLR levels. Elevated PLR levels are also reportedly associated with the progression and prognosis of many disorders, such as atherosclerosis^9,16^, and diabetes mellitus^2^. In another study, Akbas *et al*. indicated that PLR was positively associated with increased visceral fat deposition in the heart in diabetic patients^17^. Moreover, Peng *et al*. demonstrated that PLR is higher in patients with polymyositis (a chronic muscle inflammation) compared to healthy controls^18^. In the present study, we found that sarcopenia patients had higher levels of PLR than non-sarcopenia subjects and that a high PLR negatively related with the SMI, which may be attributable to the higher inflammation levels in this population.

Platelets can secrete inflammatory substances and interact with various cell types, including dendritic cells, endothelial cells, neutrophils, T-lymphocytes, and mononuclear phagocytes. The interactions between platelets and these cells may initiate or exacerbate the inflammation of the arterial wall^19^. Platelet-monocyte interaction promotes the levels of circulating monocytes with a pro-inflammatory phenotype, which have a higher affinity for adhesion to the endothelium^20^. This mechanism is supported by monocytes in the vessel wall that promote the development of atherosclerosis^21^. Platelets also contain vitamin D receptors and Stach *et al*. found that 1α,25-Dihydroxyvitamin D3 attenuates platelet activation, which could have early therapeutic relevance in atherosclerotic disease^22^. Moreover, a high platelet count is a sign of prothrombotic status and ongoing inflammation^8^. Thus, atherosclerosis and sarcopenia may both facilitate the development of abnormalities and share a common pathway^23^. In a study on the association between thigh muscle mass, carotid intima-media thickness, and brachial-ankle pulse in 496 middle-aged to elderly individuals, researchers found that arterial stiffness is associated with lower thigh muscle mass in middle-aged to elderly men^23^. In fact, the risk factors of sarcopenia, such as advanced age and low physical activity, are also related to atherosclerosis^24^. A high platelet count indicates ongoing inflammation and may affect the development of sarcopenia.

Platelets also play a role in bone formation and resorption, and may be associated with osteoporosis^25^. Platelets have vitamin D receptors that are involved in bone remodeling^26^. Increased megakaryocyte counts lead to changes in osteoclast and osteoblast function^27^, whereas changes in megakaryocyte counts may also be related to platelet number and size. Rizzoli *et al*. described the importance of sufficient vitamin D levels for bone and muscle health^28^. Accumulating evidence suggests that osteoporosis and sarcopenia may share many common pathways in the reduction of physical activity^29^. Study had shown that platelets not only interact with endothelial cells and leukocytes, but also release inflammatory substances^30^. These results indicate that increased platelets affect inflammatory status and, in turn, the skeletal muscle or bone cells, which are the main sites of sarcopenia and osteoporosis, respectively.

In particular, low lymphocyte counts, which represent a suppressed immune response and systemic inflammatory response, have also been associated with arthrosclerosis, cardiovascular disease^31^ and type 2 diabetes^32^. Age-associated changes in the immune system contribute to the high susceptibility of elderly individuals to infectious diseases, and possibly to cancer and autoimmune disorders^33^. Furthermore, low lymphocyte counts are related to an increased mortality risk in community-dwelling residents aged ≥85 years without any apparent disease^34^. Therefore, a low lymphocyte count is an indicator of a general decline in physiological function^35^ that may eventually lead to mortality.

The major findings of the present study are as follows: there is a significant positive correlation between PLR levels and sarcopenia severity, as assessed by the bioelectrical impedance analysis equation of Janssen *et al*. in elderly individuals^36^. There was a positive correlation between PLR levels and the levels of other systemic inflammatory markers (such as CRP) in patients with sarcopenia; and PLR levels exhibit an independent negative association with the SMI and positive association with sarcopenia on multivariate regression analysis after adjusting for other conventional risk factors.

The cross-sectional nature of the study is a primary limitation, which may have led to measurement, selection, and recall bias. Second, this is cross-sectional study and may not demonstrate a cause-and-effect relationship. Third, data on potential confounding factors, such as IL-6, IL-18, TNF-α, and myostatin were not available in the NHANES III study. Forth, the methodology used to classify sarcopenia status using the SMI has not been validated. Moreover, different automated hematological analysers can also yield varying neutrophil, lymphocyte, and platelet counts^37^. Furthermore, there is currently no established cut-off value for PLR level. Gary *et al*. indicated that PLR >150 could serve as a reference value in patients with peripheral arterial occlusive disease-causing limb ischemia^16^. However, in patients with sarcopenia, no such accepted PLR values are available.

We found that PLR is independently associated with sarcopenia. PLR is an inexpensive and objective parameter that can be determined from routine blood tests. Regular follow-up of PLR can aid in sarcopenia surveillance. Further prospective investigations on PLR could provide further insights its association with sarcopenia.

## Material and Methods

### Study design and participants

We enrolled adults aged ≥60 years in the NHANES III dataset. The NHANES is a health population-based survey conducted in non-institutionalized US citizens. NHANES III was conducted by the National Center for Health Statistics (NCHS) of the Centers for Disease Control and Prevention (CDC) during 1988–1994. Trained examiners collected information from participants during the home interview, including age, sex, race, medical history, standard medical examinations, and the results of the physical examinations. The dataset can be downloaded and analysed from the NHANES website, and is accessible without any permission. All the study participants signed informed consent forms prior to participation, and the Research Ethics Review Board of the National Center for Health Statistics approved the study.

### Body composition

Body composition data and resistance values (ohms, Ω) from bioelectrical impedance analysis were obtained using Valhalla 1990B Bio-Resistance Body Composition Analyzer (Valhalla Scientific, San Diego, CA, USA) with an operating frequency of 50 kHz at 800 μA. After completing a minimum 6-hour fast, whole-body bioelectrical impedance analysis measurements were obtained between the right wrist and ankle, while the subject was in the supine position. Height and weight were measured using a stadiometer after deep inhalation and an electronic digital scale calibrated in kilograms, respectively. All values were certified in the NHANES III.

### Sarcopenia assessment and classification

We estimated the skeletal muscle mass using the bioelectrical impedance analysis equation of Janssen *et al*.^36^, which has been validated using skeletal muscle mass values obtained via magnetic resonance imaging and is shown below:

$$\begin{array}{rcl}{\rm{S}}{\rm{k}}{\rm{e}}{\rm{l}}{\rm{e}}{\rm{t}}{\rm{a}}{\rm{l}}\,{\rm{m}}{\rm{u}}{\rm{s}}{\rm{c}}{\rm{l}}{\rm{e}}\,{\rm{m}}{\rm{a}}{\rm{s}}{\rm{s}}({\rm{k}}{\rm{g}}) & = & [({\rm{h}}{\rm{e}}{\rm{i}}{\rm{g}}{\rm{h}}{\rm{t}}\,{({\rm{c}}{\rm{m}})}^{2}/{\rm{b}}{\rm{i}}{\rm{o}}{\rm{e}}{\rm{l}}{\rm{e}}{\rm{c}}{\rm{t}}{\rm{r}}{\rm{i}}{\rm{c}}{\rm{a}}{\rm{l}}\,{\rm{i}}{\rm{m}}{\rm{p}}{\rm{e}}{\rm{d}}{\rm{a}}{\rm{n}}{\rm{c}}{\rm{e}}\,{\rm{a}}{\rm{n}}{\rm{a}}{\rm{l}}{\rm{y}}{\rm{s}}{\rm{i}}{\rm{s}}\,{\rm{r}}{\rm{e}}{\rm{s}}{\rm{i}}{\rm{s}}{\rm{t}}{\rm{a}}{\rm{n}}{\rm{c}}{\rm{e}}\,({\rm{\Omega }})\\  &  & \times 0.401)+({\rm{s}}{\rm{e}}{\rm{x}}\,({\rm{w}}{\rm{o}}{\rm{m}}{\rm{e}}{\rm{n}}=0;{\rm{m}}{\rm{e}}{\rm{n}}=1)\times 3.825)\\  &  & +\,({\rm{a}}{\rm{g}}{\rm{e}}\,({\rm{y}}{\rm{e}}{\rm{a}}{\rm{r}}{\rm{s}})\times \mbox{--}0.071)]+\mathrm{5.102.}\end{array}$$

The SMI was estimated as the skeletal muscle mass adjusted for height squared (m^2^). We used the sex-specific cutoffs proposed by Janssen *et al*.^38^ based on the risk of physical disability. The sarcopenia severity was classified as normal (≥10.76 kg/m^2^), class I (8.51–10.75 kg/m^2^), and class II (≤8.50 kg/m^2^) in men; and normal (≥6.76 kg/m^2^), class I (5.76–6.75 kg/m^2^), and class II (≤5.75 kg/m^2^) in women. Therefore, patients with sarcopenia were classified as class I or II.

### Measurement of PLR

Blood was collected from all subjects; patients with haemophilia or chemotherapy within the last 4 weeks were excluded^39^. CBCs were obtained from participants in the NHANES using the Beckman Coulter method^40^; in particular, a quantitative, automated, differential cell counter (Coulter Counter Model S-PLUS JR [Beckman Coulter Inc., Fullerton, CA]) was used to calculate the exact values for hematological measurements and provide information on the platelet count, and absolute neutrophil and lymphocyte counts. PLR and neutrophil-to-lymphocyte ratio (NLR) values were estimated as the ratio of the platelet count to the lymphocyte cell count and the neutrophil cell count to the lymphocyte cell count, respectively.

### Covariates

The physical performance examination was performed at a mobile examination center or in the participants’ home if they were unable to visit the examination center. Every participant completed 2 trials of an 8-foot walk at his/her usual walking pace, with the use of assistive devices allowed, but not another person’s assistance. The time in seconds to complete each task was recorded by a technician^41^. Gait speed was calculated as walking distance (8 feet = 2.44 m), divided by time (seconds). Self-reported data, including age, sex, race/ethnicity, smoking history, and medical history were obtained. Subjects were asked the question “Do you now smoke cigarettes?” to determine the smoking status. Participants were considered to have heart disease if they had been diagnosed with the condition or had experienced a heart attack. The presence of stroke was self-reported by the subjects. Diabetes was considered based on self-reported doctor’s diagnosis or random glucose level ≥200 mg/dL, fasting glucose level ≥126 mg/dL, or anti-diabetes medication use. Blood pressure was determined in the right arm, unless the participants had a specific condition. Waist circumference was determined to the nearest 0.1 cm at the high point of the iliac crest, during minimal respiration. Chemical analyses of serum biochemical profiles were performed at the Lipoprotein Analytical Laboratory at Johns Hopkins University, Baltimore, Maryland. Uric acid was measured enzymatically using a Hitachi 737 automated multichannel chemistry analyser (Boehringer Mannheim Diagnostics, Indianapolis, IN, USA). Serum glucose levels were ascertained using an enzymatic assay (CobasMira assay). CRP levels were determined using a Behring latex-enhanced assay, and were quantified using latex-enhanced nephelometry with the Behring Nephelometer Analyzer System (Behring Diagnostics, Inc, Somerville, NJ). Plasma fibrinogen levels were measured from blood plasma by using the Clauss clotting method. Serum 25 (OH) D was measured at the NCH, CDC, Atlanta, GA by using a radioimmunoassay kit (Diasorin Inc., Stillwater MN, USA). Consistent with the revised National Cholesterol Education Program’s Adult Treatment Panel III (NCEP: ATP III), MetS was defined as the presence of ≥3 of the following characteristics: abdominal obesity, with waist circumference ≥102 cm in men and ≥88 cm in women; high triglyceride levels (≥150 mg/dL) in patients not currently using lipid-lowering medications; low HDL levels of <40 mg/dL in men and <50 mg/dL in women or patients receiving specific drug treatment; high blood pressure, with systolic blood pressure ≥130 mmHg, diastolic blood pressure ≥85 mmHg, or the current use of antihypertensive drugs; and high fasting glucose levels (≥110 mg/dL) or current use of insulin or oral diabetic medications^42^.

All the protocols used standardized methods with documented accuracy with respect to the CDC reference methods. Detailed specimen collection information is available at the NHANES website. The National Center for Health Statistics Institutional Review Board (IRB) approved the study protocol. Our analysis exclusively used de-identified data, and hence, it did not require a review from the IRB.

### Statistical analyses

SPSS (v18.0 for Windows; SPSS Inc., Chicago, IL, USA) was used to conduct statistical analyses. The data are expressed as mean with SD or median value with the interquartile range (IQR). The Chi-square test was used for analysing categorical data, whereas analysis of variance (ANOVA) with the Kruskal-Wallis test was used for analysing continuous data. Two-sided p-values of <0.05 were considered significant. Thereafter, the associations between PLR and sarcopenia severity were determined. We divided the serum PLR levels into quartiles, and the subjects in the lowest quartile group were used as the reference; the cut-off levels for serum PLR quartiles were: Q1, >95; Q2, 95 to <120; Q3, 120 to <155; and Q4, ≥155. As mentioned earlier, sarcopenia was classified as either class I or II. The OR for sarcopenia was determined using multiple logistic regression analysis, by comparing each subject in the upper 3 serum PLR level quartiles to the subjects in the lowest quartile. Based on previous studies, demographic factors that influence the clinical findings were adjusted for and determined as covariates. In particular, the extended model approach was used for covariate adjustment: Model 1 = age, sex, race; Model 2 = Model 1 + chronic diseases (MetS, stroke, and heart diseases) + health behaviours (current smoker); Model 3 = Model 2 + CRP, fibrinogen, UA, total bilirubin levels, creatinine, albumin and vitamin D, and Model 4 = Model 3 + Gait speed. Trend tests were conducted by treating the quartiles of serum PLR levels from Q1 to Q4 as continuous variables, in order to observe the association of the OR of sarcopenia across increasing quartiles of serum PLR.

### Data availability statement

The datasets generated during and/or analysed during the current study are available from the corresponding author on reasonable request.

### Ethics statement

The National Center for Health Statistics Institutional Review Board approved the NHANES III study. All participants wrote informed consent before the study. We analysed an openly unidentifiable, available online database. Therefore, this study was exempt from IRB review. All methods were performed based on the relevant guidelines.
